# Supplementary material for: What determines the information update rate in echolocating bats
Source: Commun Biol. 2023 Nov 22;6:1187. doi: 10.1038/s42003-023-05563-x (PMC10663583; doi:10.1038/s42003-023-05563-x)
Supplement: Supplementary file 2 — Supplementary material [file 42003_2023_5563_MOESM2_ESM.pdf]

Supplementary material for:

What determines the information update rate in echolocating bats

Authors: Mor Taub[1,8], Aya Goldshtein[1,5,6,7,8], Arjan Boonman[1], Ofri Eitan[1], Edward Hurme[2,3], Stefan Greif[1] and Yossi Yovel[1,4*]

1. School of Zoology, Faculty of Life Sciences, Tel Aviv University, Tel Aviv 6997801, Israel.
2. Department of Migration, Max Planck Institute of Animal Behavior, Radolfzell, Germany
3. Centre for the Advanced Study of Collective Behaviour, University of Konstanz, Konstanz, Germany
4. Sagol School of Neuroscience, Tel Aviv University, Tel Aviv 6997801, Israel
5. Present address: Department of Collective Behaviour, Max Planck Institute of Animal Behaviour, Konstanz 78464, Germany.
6. Present address: Centre for the Advanced Study of Collective Behaviour, University of Konstanz, Konstanz, Germany
7. Present address: Department of Biology, University of Konstanz, Konstanz, Germany
8. These authors contributed equally: Mor Taub, Aya Goldshtein.

***Corresponding Author**:

Yossi Yovel, E-mail: yossiyovel@gmail.com.

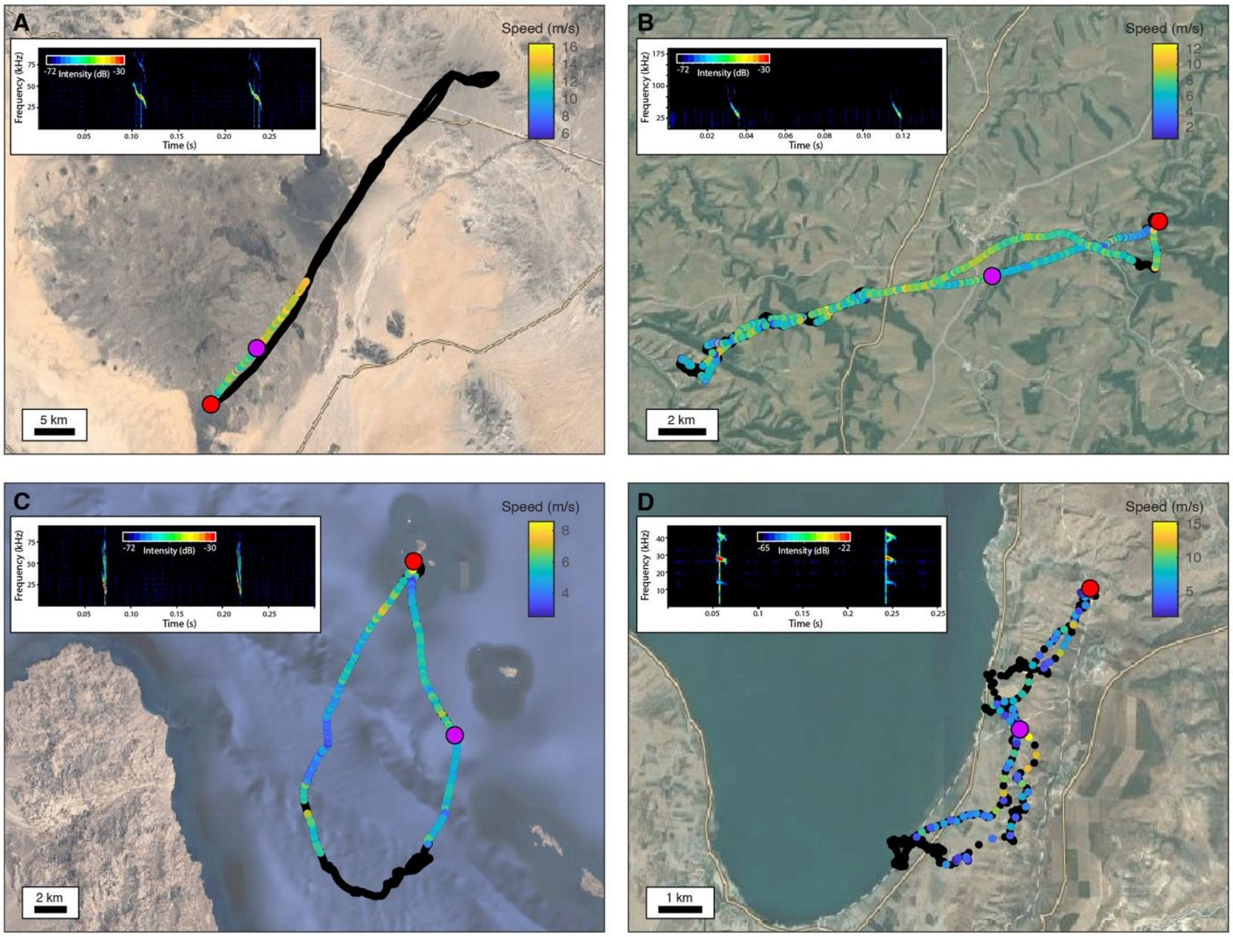

Supplementary Figure 1: **Examples of bats' flight trajectories and echolocation calls**. Bats' flight trajectories of one individual of (**A**) *L. yerbabuenae*, (**B**) *M. myotis*, (**C**) *M. vivesi,* and (**D**) *R. microphyllum* are color-coded according to their ground speed (blue to yellow scale). Black dots represent GPS positions without analyzed audio data. The colonies of the bats are presented by red circles. Inset panels show examples of bats' echolocation calls, with corresponding recording locations marked by purple circles.

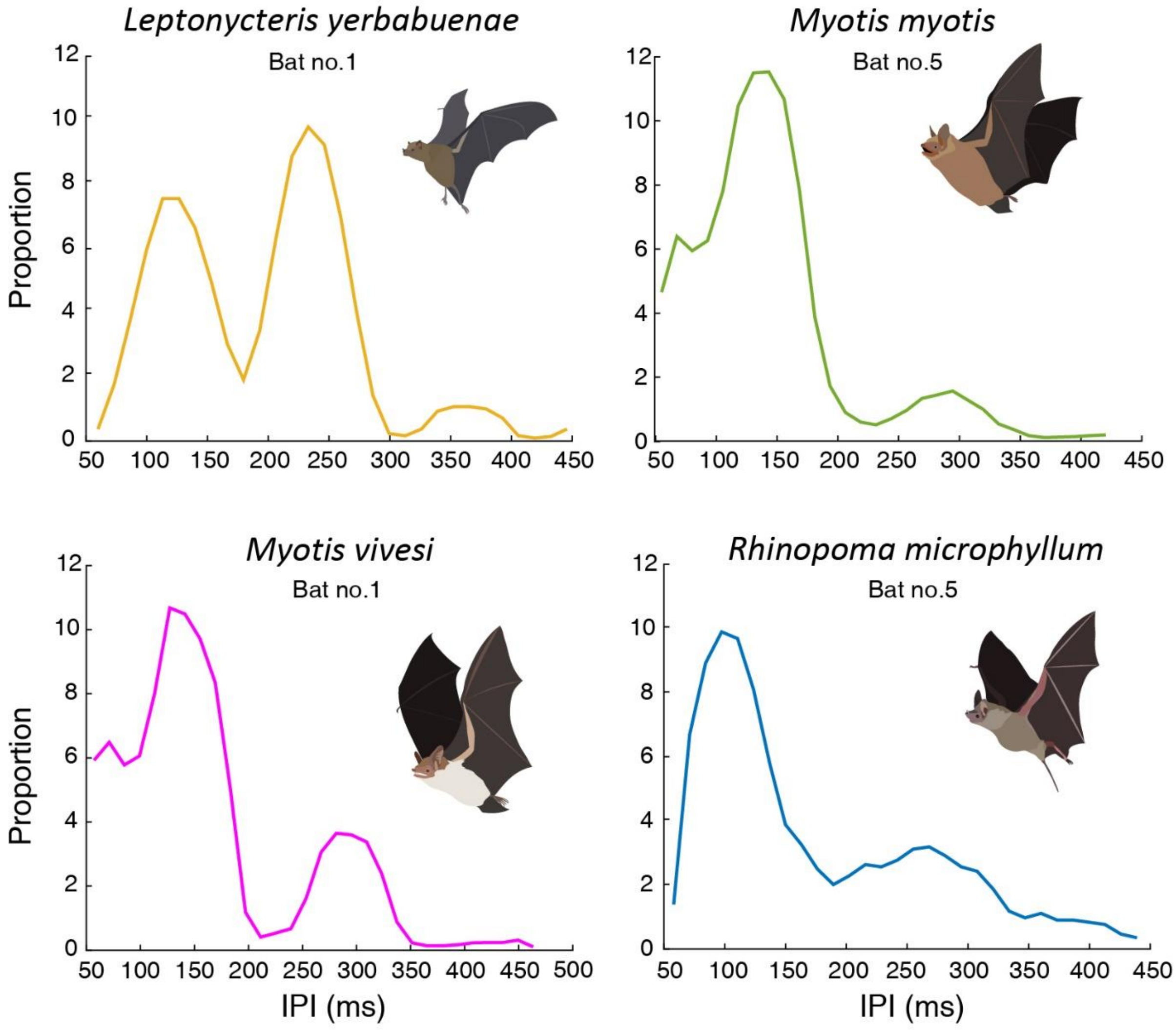

Supplementary Figure 2: IPI distribution of individual bats. An example of one individual is shown for each species.

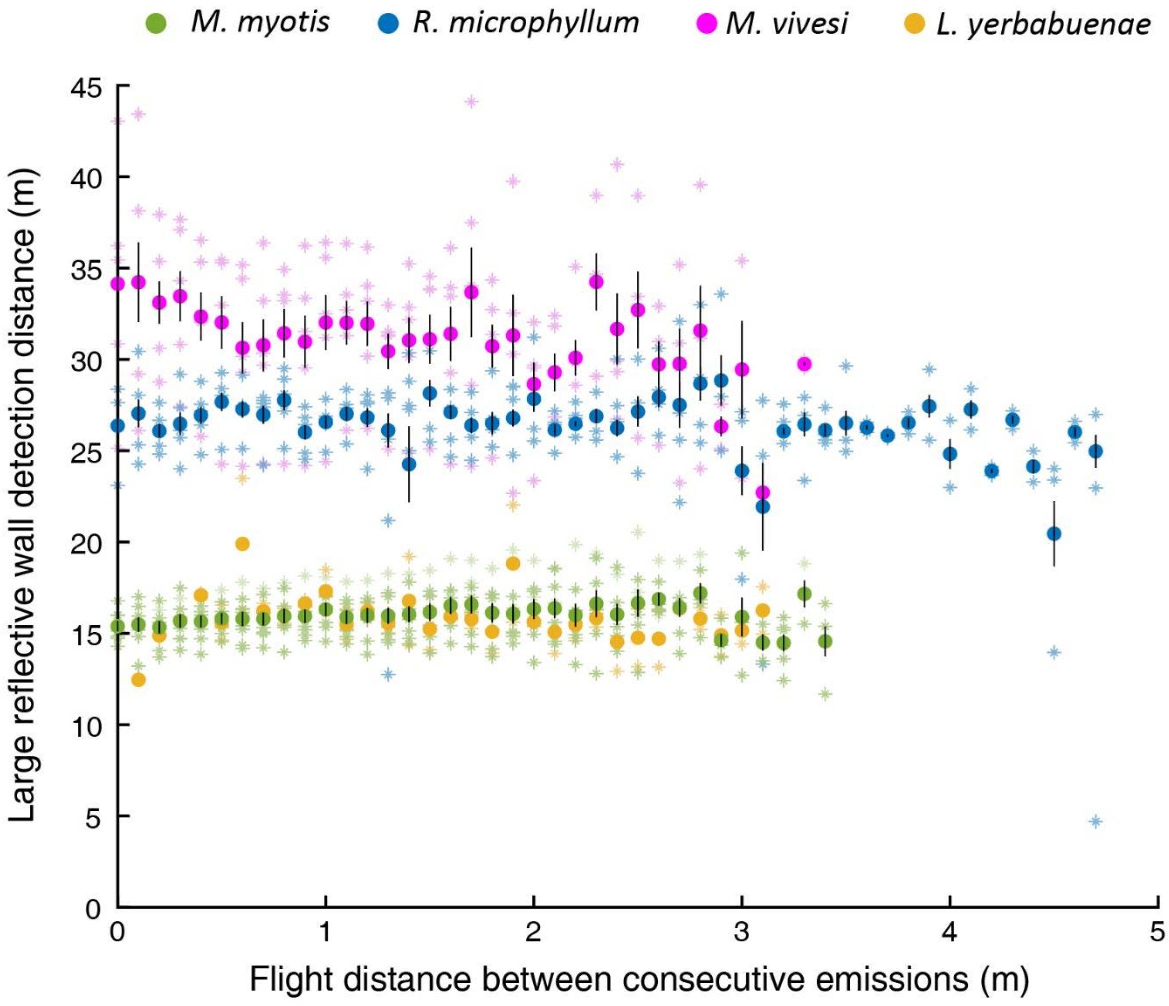

Supplementary Figure 3: Large reflective wall detection range. Large reflective wall detection distance compared to flight distance of the four bat species. Circles represent the average detection distance estimated for different flight distances based on the actual signal parameters and the flight speed. Asterisks represent the individual data points for each species. Error bars depict the SE. N=22 bats from all four species in total.

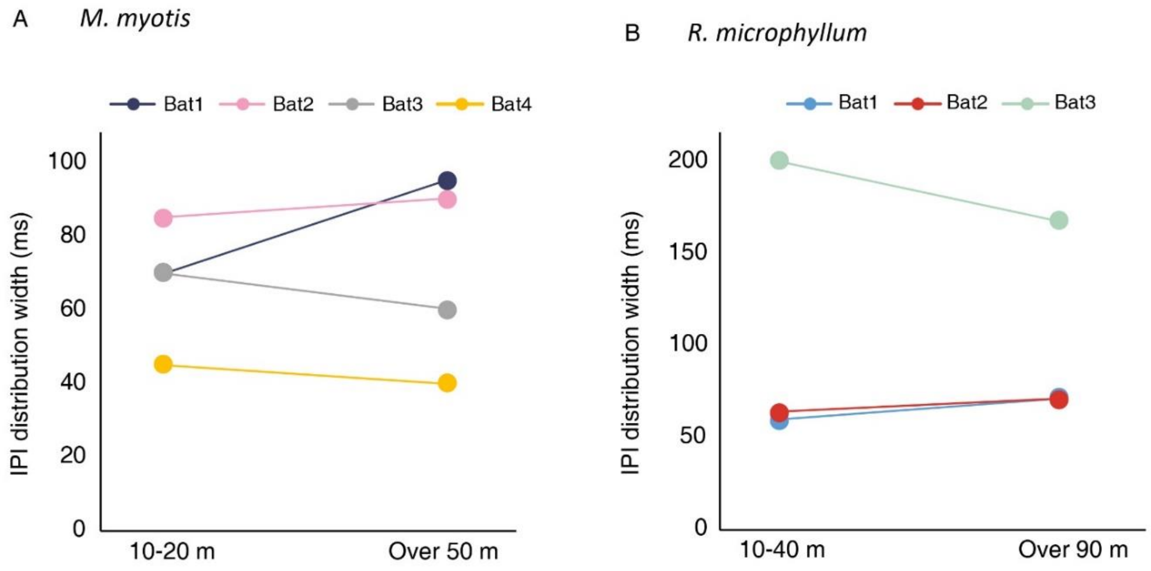

Supplementary Figure 4: IPI distribution width in different altitude ranges. **(A)** IPI distribution width of individual *M. myotis* bats at commute flights of 10-20 m and over 50 m. **(B)** IPI distribution width of individual *R. microphyllum* bats at commute flights of 10-40 m and over 90 m.

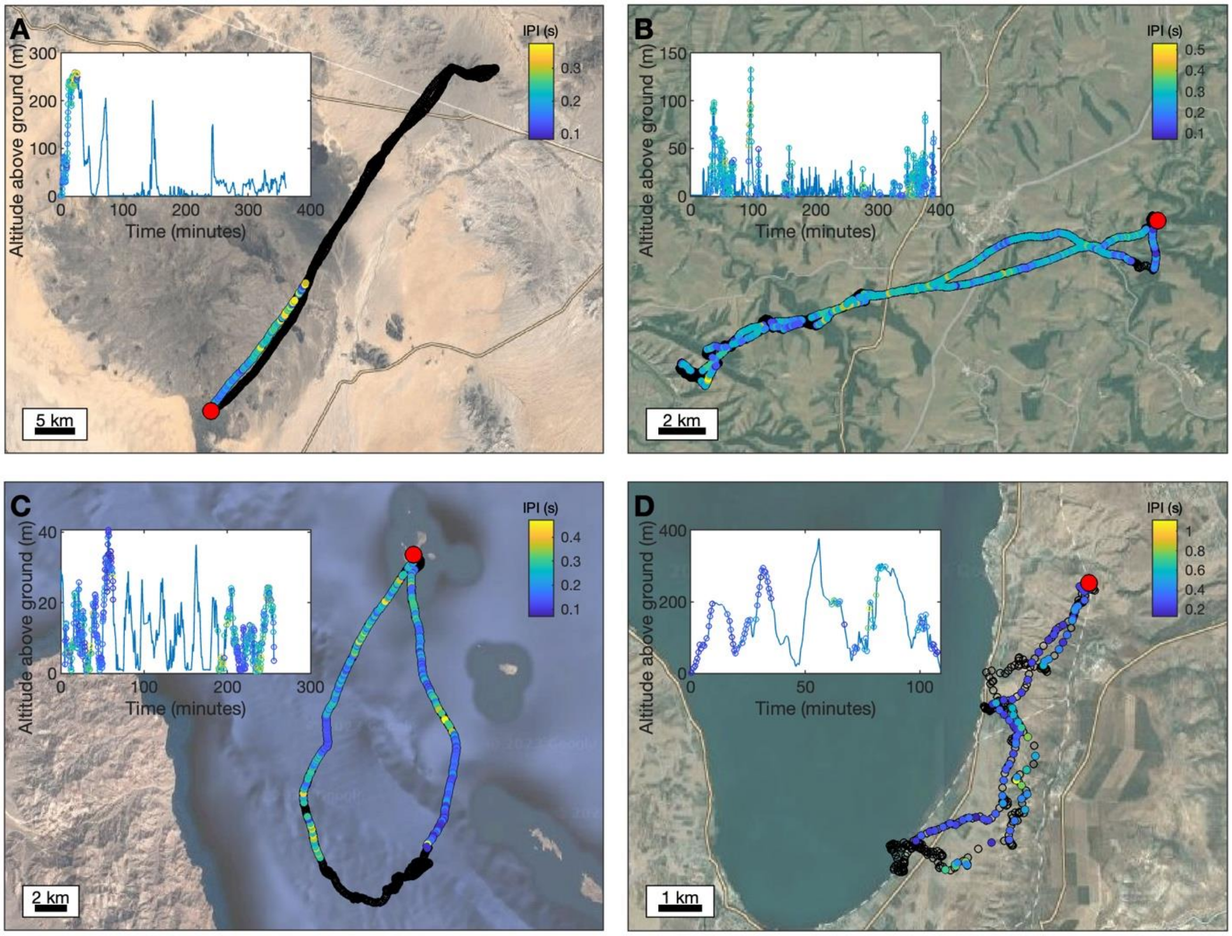

Supplementary Figure 5: **Examples of bats' IPI by altitude along the flight trajectories**. Bats' flight trajectories and flight altitude above ground (insert panels) of one individual of (**A**) *L. yerbabuenae*, (**B**) *M. myotis*, (**C**) *M. vivesi,* and (**D**) *R. microphyllum* are color-coded according to their IPI (blue to yellow scale). Black dots represent GPS positions without analyzed audio data. The colonies of the bats are presented by red circles.

Supplementary Table 1. Direction of influence of the different parameters on the IPI by species.

**Parameter**	*Leptonycteris yerbabuenae*	*Myotis myotis*	*Myotis vivesi*	*Rhinopoma microphyllum*
Peak-energy frequency (kHz)	-	-	-	-
Intensity (dB)	NA	-	-	-
Speed ($m \cdot s^{-1}$)	NA	+	+	+
Altitude (m)	+	+	-	NA
