# Supplementary material for: What determines the information update rate in echolocating bats
Source: Commun Biol. 2023 Nov 22;6:1187. doi: 10.1038/s42003-023-05563-x (PMC10663583; doi:10.1038/s42003-023-05563-x)
Supplement: Supplementary file 3 — Reporting Summary [file 42003_2023_5563_MOESM3_ESM.pdf]

nature portfolio

Corresponding author(s): Mor Taub

Last updated by author(s): Oct 25, 2023

Reporting Summary

Nature Portfolio wishes to improve the reproducibility of the work that we publish. This form provides structure for consistency and transparency in reporting. For further information on Nature Portfolio policies, see our Editorial Policies and the Editorial Policy Checklist.

Statistics

For all statistical analyses, confirm that the following items are present in the figure legend, table legend, main text, or Methods section.

n/a	Confirmed	
☐	☒	The exact sample size (*n*) for each experimental group/condition, given as a discrete number and unit of measurement
☐	☒	A statement on whether measurements were taken from distinct samples or whether the same sample was measured repeatedly
☐	☒	The statistical test(s) used AND whether they are one- or two-sided<br>*Only common tests should be described solely by name; describe more complex techniques in the Methods section.*
☐	☒	A description of all covariates tested
☐	☒	A description of any assumptions or corrections, such as tests of normality and adjustment for multiple comparisons
☐	☒	A full description of the statistical parameters including central tendency (e.g. means) or other basic estimates (e.g. regression coefficient) AND variation (e.g. standard deviation) or associated estimates of uncertainty (e.g. confidence intervals)
☐	☒	For null hypothesis testing, the test statistic (e.g. F, t, r) with confidence intervals, effect sizes, degrees of freedom and P value noted<br>*Give P values as exact values whenever suitable.*
☒	☐	For Bayesian analysis, information on the choice of priors and Markov chain Monte Carlo settings
☒	☐	For hierarchical and complex designs, identification of the appropriate level for tests and full reporting of outcomes
☐	☒	Estimates of effect sizes (e.g. Cohen's d, Pearson's r), indicating how they were calculated

Our web collection on statistics for biologists contains articles on many of the points above.

Software and code

Policy information about availability of computer code

Data collection	Data collection was performed with on board GPS devices: Robin, Lucid Ltd., Israel or Vesper, ASD inc., Israel
Data analysis	Data analysis was performed in MATLAB and JMP pro (statistical analysis). MATLAB code will be available in Mendeley data repository as stated in the manuscript

For manuscripts utilizing custom algorithms or software that are central to the research but not yet described in published literature, software must be made available to editors and reviewers. We strongly encourage code deposition in a community repository (e.g. GitHub). See the Nature Portfolio guidelines for submitting code & software for further information.

Data

Policy information about availability of data

All manuscripts must include a data availability statement. This statement should provide the following information, where applicable:

- Accession codes, unique identifiers, or web links for publicly available datasets
- A description of any restrictions on data availability
- For clinical datasets or third party data, please ensure that the statement adheres to our policy

The datasets generated and analyzed during the current study are available on Mendeley Data: Doi: 10.17632/w4s2xrkv6p.1

Research involving human participants, their data, or biological material

Policy information about studies with human participants or human data. See also policy information about sex, gender (identity/presentation), and sexual orientation and race, ethnicity and racism.

Reporting on sex and gender	*Use the terms sex (biological attribute) and gender (shaped by social and cultural circumstances) carefully in order to avoid confusing both terms. Indicate if findings apply to only one sex or gender; describe whether sex and gender were considered in study design; whether sex and/or gender was determined based on self-reporting or assigned and methods used. Provide in the source data disaggregated sex and gender data, where this information has been collected, and if consent has been obtained for sharing of individual-level data; provide overall numbers in this Reporting Summary. Please state if this information has not been collected. Report sex- and gender-based analyses where performed, justify reasons for lack of sex- and gender-based analysis.*
Reporting on race, ethnicity, or other socially relevant groupings	*Please specify the socially constructed or socially relevant categorization variable(s) used in your manuscript and explain why they were used. Please note that such variables should not be used as proxies for other socially constructed/relevant variables (for example, race or ethnicity should not be used as a proxy for socioeconomic status). Provide clear definitions of the relevant terms used, how they were provided (by the participants/respondents, the researchers, or third parties), and the method(s) used to classify people into the different categories (e.g. self-report, census or administrative data, social media data, etc.) Please provide details about how you controlled for confounding variables in your analyses.*
Population characteristics	*Describe the covariate-relevant population characteristics of the human research participants (e.g. age, genotypic information, past and current diagnosis and treatment categories). If you filled out the behavioural & social sciences study design questions and have nothing to add here, write "See above."*
Recruitment	*Describe how participants were recruited. Outline any potential self-selection bias or other biases that may be present and how these are likely to impact results.*
Ethics oversight	*Identify the organization(s) that approved the study protocol.*

Note that full information on the approval of the study protocol must also be provided in the manuscript.

Field-specific reporting

Please select the one below that is the best fit for your research. If you are not sure, read the appropriate sections before making your selection.

☐ Life sciences ☐ Behavioural & social sciences ☒ Ecological, evolutionary & environmental sciences

For a reference copy of the document with all sections, see nature.com/documents/nr-reporting-summary-flat.pdf

Ecological, evolutionary & environmental sciences study design

All studies must disclose on these points even when the disclosure is negative.

Study description	The study explores the impact of different echolocation and movement parameters on the sampling rate of four species of echolocating bats that differ in their foraging strategies. In this study we used on-board recordings to reveal how these species regulate sensory acquisition rate during commute flights.
Research sample	A total of 25 bats participated in data collection. Leptonycteris yerbabuenae: n=5; Myotis myotis: n=8; Myotis vivesi: n=6 and Rhinopoma microphyllum: n=6.
Sampling strategy	Sample size was limited by the successful retrieval of data from GPS devices.
Data collection	We tracked bats' movement and echolocation using a miniature GPS device (Robin, Lucid Ltd., Israel or Vesper, ASD inc., Israel) with an on-board ultrasonic microphone (FG-23329, Knowles) that was attached to a telemetry unit (LB-2X 0.3 g, Holohil Systems Ltd., Canada). The devices were wrapped with waterproof balloons and were mounted on the back of the bats using surgical cement (Perma-Type, McKesson Patient Care Solutions, Inc., USA). The bats were held for a few minutes to allow the glue to dry properly, then rested for another 15 minutes in a fabric bag and released at the same location. The devices were located using telemetry a few days later, and retrieved after the device fell off the bat or gently removed from the recaptured bat.
Timing and spatial scale	Myotis vivesi: data was collected in May-June 2015; Myotis myotis: data was collected in July-August 2013; Leptonycteris yerbabuenae: data was collected in May-June 2015 and Rhinopoma microphyllum: data was collected in August 2012.
Data exclusions	NA
Reproducibility	Reproducibility is possible with similar devices.
Randomization	NA

Blinding	Blinding was not possible because of specific acoustic features of each species

Did the study involve field work? ☒ Yes ☐ No

Field work, collection and transport

Field conditions	NA
Location	Myotis vivesi: Partida Norte Island, Mexico (28 52 30’’N, 113 2 17’’W); Leptonycteris yerbabuenae: El Pinacate y Gran Desierto de Altar Biosphere Reserve, Sonoran Desert, Mexico. Myotis myotis: Orlova Chuka cave in northeastern Bulgaria; Rhinopoma microphyllum: Northern Israel.
Access & import/export	Animals were captured at their roosts and shortly after released at the same location. Animal capture in Bulgaria, Mexico and Israel were conducted under permits of the responsible authorities: permits #2011/38346 and 2012/38346 from the NPA, and #L-11-054 from the Tel-Aviv University IACUC, Israel; L. yerbabuenae (Mexico): permit #04019/15, 03946/15 14509/16 from Dirección General de Vida Silvestre; M.myotis (Bulgaria): MOEW-Sofia and RIOSV-Ruse, Bulgaria, permit #465/29.06.2012 and 639/28.05.2015. M. vivesi (Mexico): permits #7668-15 and 2492-17 from Dirección General de Vida Silvestre, and permits # 17-16 and 21-17 from Secretaría de Gobernación.
Disturbance	Animals were captured at their roosts and shortly after released at the same location at minimum disturbance to the roost.

Reporting for specific materials, systems and methods

We require information from authors about some types of materials, experimental systems and methods used in many studies. Here, indicate whether each material, system or method listed is relevant to your study. If you are not sure if a list item applies to your research, read the appropriate section before selecting a response.

Materials & experimental systems

n/a	Involved in the study	
☒	☐	Antibodies
☒	☐	Eukaryotic cell lines
☒	☐	Palaeontology and archaeology
☐	☒	Animals and other organisms
☒	☐	Clinical data
☒	☐	Dual use research of concern
☒	☐	Plants

Methods

n/a	Involved in the study	
☒	☐	ChIP-seq
☒	☐	Flow cytometry
☒	☐	MRI-based neuroimaging

Animals and other research organisms

Policy information about studies involving animals; ARRIVE guidelines recommended for reporting animal research, and Sex and Gender in Research

Laboratory animals	NA
Wild animals	Bats from four species: Leptonycteris yerbabuenae (Phyllostomidae), Myotis myotis (Vespertilionidae), Myotis vivesi (Vespertilionidae), and Rhinopoma microphyllum (Rhinopomatidae) were captured next to their roost using a mist net, hand net, or gloved hand and fitted with a miniature GPS device using surgical cement. The bats were held for a few minutes to allow the glue to dry properly, then rested for another 15 minutes in a fabric bag and released at the same location.
Reporting on sex	Data was collected from lactating or post lactating females
Field-collected samples	NA
Ethics oversight	All experiments were conducted according to the following permits of the responsible authorities: R. microphyllum (Israel): permits #2011/38346 and 2012/38346 from the NPA, and #L-11-054 from the Tel-Aviv University IACUC, Israel; L. yerbabuenae (Mexico): permit #04019/15, 03946/15 14509/16 from Dirección General de Vida Silvestre; M.myotis (Bulgaria): MOEW-Sofia and RIOSV-Ruse, Bulgaria, permit #465/29.06.2012 and 639/28.05.2015. M. vivesi (Mexico): permits #7668-15 and 2492-17 from Dirección General de Vida Silvestre, and permits # 17-16 and 21-17 from Secretaría de Gobernación.

Note that full information on the approval of the study protocol must also be provided in the manuscript.
